# Coping with Fungal Diseases in Crops: New Advances in Genomics, Breeding and Management

**DOI:** 10.3390/genes14091758

**Published:** 2023-09-04

**Authors:** Elisabetta Mazzucotelli, Anna Maria Mastrangelo

**Affiliations:** 1 Council for Agricultural Research and Economics, Research Centre for Genomics and Bioinfomatics, 29017 Fiorenzuola d’Arda, Italy; elisabetta.mazzucotelli@crea.gov.it; 2 Council for Agricultural Research and Economics, Research Centre for Cereal and Industrial Crops, 71122 Foggia, Italy

This Special Issue comprises a collection of eight peer-reviewed articles centered around the plant–pathogen interaction with the aim of proposing strategies that enhance plant resistance to pathogens and limit the damage to crop production, utilizing a multidisciplinary approach. A total of 67 authors contributed to this collection. Among the accepted submissions, the following topics were covered: quantitative trait loci (QTL) mapping (three articles), molecular and physiological responses to pathogen infection (two articles), phenotypic evaluation to assess the reaction of germplasm collections expressing crop biodiversity to pathogen infection (two articles), and the population genetics of plant pathogens (one article).

Agricultural systems are characterized by a continuous plant–pathogen co-evolution, and the emergence of new virulent races is amplified by the current climate changes. Hence, identifying new sources of resistance to plant pathogens is of paramount importance to protect crop yield and quality while concurrently reducing the use of pesticides and increasing agricultural sustainability. Therefore, the phenotypic characterization of extensive collections of lines in response to different pathogens is a vital approach to identify new sources of resistance.

In this regard, Ben M’Barek et al. [1] evaluated a collection of 1059 Mediterranean durum wheat landraces from 13 countries to determine their resistance levels to the *Zymoseptoria tritici* fungus at the seedling and adult stages in Tunisia, which is known as a hotspot for this disease. The results revealed a high frequency of resistance reactions, with more than 50% of lines exhibiting an immune reaction at both seedling and adult growth stages. 

In a similar approach, Laribi et al. [2] evaluated a Mediterranean collection of 549 durum wheat accessions under field conditions to determine their resistance levels to tan spot induced by the *Pyrenophora tritici-repentis* (*Ptr*) fungus. The assessment spanned two cropping seasons in Tunisia, a hotspot for *Ptr*. Nearly 50% of the accessions displayed promising resistance levels, suggesting their potential as diverse resistance sources to *Ptr* that can be incorporated into breeding programs.

Both studies also emphasized the significance of evaluating plant height, which was observed to exhibit a negative effect on adult-stage resistance to *Zymoseptoria tritici*, while displaying a negative correlation with *Ptr* disease scores. These results indicate that plant height may either significantly influence tan spot severity or potentially serve as disease-escape trait.

Once identified, resistant varieties can be immediately recommended for cultivation in regions where their genetic resistance can limit the development of the pathogen, or these varieties, along with non-adapted lines (i.e., landraces), can be employed to genetically map the resistance gene(s) and transfer them to susceptible élites. A common approach to map new resistant determinants in crops is based on the use of recombinant inbred line (RIL) populations.

Marone et al. [3] identified 14 QTLs to determine their resistance to *Puccinia graminis* f. sp. *tritici* (*Pgt*) in durum wheat. *Pgt* is one of the most destructive fungal diseases affecting durum and common wheat worldwide; therefore, the identification of new resistant genes/alleles is of significant importance to balance the continuous plant–pathogen co-evolution. The QTLs were mapped to chromosomes 3A, 4A, 6A, and 6B in four RIL populations, and the reaction of parents to different *Pgt* races suggests that novel genes or alleles could be present on chromosomes 3A and 6B. Moreover, because of the information available from the reference genome of durum cv. ‘Svevo’, putative candidate genes with a disease-related functional annotation have been identified in the QTL regions.

Bariana et al. [4] used a similar approach by analyzing a RIL population of common wheat derived from the Aus27352/Avocet cross to identify the *Lr82* gene on the long arm of chromosome 2B, which promotes resistance to leaf rust. The KASP_22131 marker, mapped 0.8 cm proximal to *Lr82*, was developed to facilitate the marker-assisted pyramiding of *Lr82* in breeding programs, following a polymorphism check on the parental lines.

Langlands-Perry et al. [5] studied the quantitative resistance to *Septoria tritici* blotch (STB) in common wheat using a population derived from a cross between the French cultivar ‘Renan’, which is generally resistant to STB, and the susceptible cv. ‘Chinese Spring’. Quantitative resistance is considered more durable than qualitative resistance, as it does not involve major resistance genes that can be easily overcome by pathogen populations; rather, it involves a combination of genes with lower individual effects. QTLs on chromosomes 7B, 1D, and 5D were identified, of which the QTL on 7B was detected for rection to two fungal strains, whereas those on 1D and 5D were strain-specific. The QTL regions included several genes coding for wall-associated kinases (WAK), nucleotide-binding and leucine-rich repeat (NB-LRR) receptors, and proteins carrying kinase domains, some of which were expressed during the infection process.

While the genetic analysis of plant–pathogen interactions is beneficial for the direct application of the identified molecular markers to breeding programs, the study of molecular and physiological responses of plants to pathogen infections enables the identification of genes that are important for resistance as promising targets for breeding initiatives. Conducting gene expression analyses on plants infected by the target pathogen is a powerful tool to identify specific genes among thousands of genes that are associated with a positive response of plants to pathogens. Mishra et al. [6] used an RNA-seq approach to study the gene expression in lentils (*Lens culinaris* Medik) after inoculation with dry root rot (*Rhizoctonia bataticola*), which is a significant disease affecting lentils. The study revealed various genes associated with changes in phenolic compounds, transcription factors, antioxidants, receptor kinases, and hormone signals, which could be involved in the reaction to dry root rot, and the study also identified some key miRNA targets.

In a forward genetic approach, the availability of mutants in specific genes is essential for demonstrating the role of these genes in plant–pathogen interactions. Wang et al. [7] used mutants in genes belonging to the ethylene pathway to investigate the molecular response of *Arabidopsis thaliana* to *Plasmodiophora brassicae* infection, which causes hypertrophy of host roots and subsequent formation of galls. EIN3/EIL1 transcription factors are the key regulators of ethylene signaling that sustain a variety of plant responses to ethylene. The study of an *ein3/eil1* double mutant provided evidence of the role of these genes, and the hormone ethylene, in increasing resistance to *P. brassicae*. This finding was further supported by the observation that the *wrky75-c* mutant of *A. thaliana*, mutated in the *WRKY75* gene directly upregulated by ethylene, was more susceptible than the wild type. 

Lastly, studies on pathogen population genetics are highly useful in understanding the diffusion and evolution of pathogen species and races and in adopting suitable solutions, such as selecting cultivars carrying specific *R* genes, to limit the impact of pathogens on crop production. Wang et al. [8] analyzed the population genetic structure of *Puccinia striiformis* f. sp. *tritici*, a causal agent of wheat stripe rust, in two provinces in China, which are significant for pathogen over-summering or over-wintering, serving as a source of inoculum in spring epidemic regions. The genetic analysis of more than 500 isolates from both locations based on simple sequence repeat markers revealed greater genetic diversification levels in the spring populations compared to the fall populations. Moreover, a small overlap of genotypes was observed, suggesting a limited genotype exchange between the populations of the two regions.

In the face of ongoing climate changes that contribute to the emergence of new virulent races, a multidisciplinary approach is crucial for driving research efforts aimed at developing plants with enhanced resistance to fungal diseases, ultimately reducing damages induced by pathogen infections and improving grain yield and quality. Genetic resistance to diseases also addresses the need to reduce pesticide use, promoting a more sustainable and environmentally friendly approach to agriculture. The articles compiled in this Special Issue demonstrate a research trajectory in which *in planta* approaches, such as identifying new sources of resistance, mapping resistant loci, and understanding the physiological and molecular mechanisms of plant responses to pathogens, are combined with the study of pathogen populations to define new strategies for enhancing disease resistance in crops.
